# Characterizing the blood–brain barrier and gut barrier with super-resolution imaging: opportunities and challenges

**DOI:** 10.1117/1.NPh.10.4.044410

**Published:** 2023-10-04

**Authors:** Ellen Doney, Renaud Bernatchez, Valérie Clavet-Fournier, Katarzyna A. Dudek, Laurence Dion-Albert, Flavie Lavoie-Cardinal, Caroline Menard

**Affiliations:** a Université Laval, Department of Psychiatry and Neuroscience, Faculty of Medicine, Quebec City, Québec, Canada; b CERVO Brain Research Center, Québec City, Québec, Canada; c Institute for Intelligence and Data, Québec City, Québec, Canada

**Keywords:** stimulated emission depletion, stochastic optical reconstruction microscopy, tight junctions, algorithms

## Abstract

Brain and gut barriers have been receiving increasing attention in health and diseases including in psychiatry. Recent studies have highlighted changes in the blood–brain barrier and gut barrier structural properties, notably a loss of tight junctions, leading to hyperpermeability, passage of inflammatory mediators, stress vulnerability, and the development of depressive behaviors. To decipher the cellular processes actively contributing to brain and gut barrier function in health and disease, scientists can take advantage of neurophotonic tools and recent advances in super-resolution microscopy techniques to complement traditional imaging approaches like confocal and electron microscopy. Here, we summarize the challenges, pros, and cons of these innovative approaches, hoping that a growing number of scientists will integrate them in their study design exploring barrier-related properties and mechanisms.

Super-resolution microscopy techniques break the diffraction limit of light, enabling the visualization and characterization of subcellular structures down to the nanoscale. In cerebrovascular research, electron microscopy (EM) has been the gold standard to visualize and quantify subcellular structures central to the brain barrier’s function, blood flow regulation, and communication within the neurovascular unit.^1^ EM first allowed for the identification of endothelial cells as the main component of the blood–brain barrier in the late 1960s.^2^^,^^3^ Utilization of horseradish peroxidase as an EM tracer revealed endothelial cells properties, such as tight junctions and a low rate of transcytosis protecting the brain from circulating harmful signals.^2^^,^^3^ Today, EM experiments are commonly performed to validate imaging results obtained with other modalities or provide additional structural information. However, EM is not very effective for the simultaneous localization of multiple proteins and tracers,^4^ and it cannot be used for *in vivo* applications.^5^ Researchers are thus turning their attention to super-resolution microscopy techniques to complement traditional approaches and gain mechanistic insights.^4^ Although a lot of neurophotonic tools have been developed to study neuron properties, functions, and interactions with other cells,^6^^–^^8^ much less attention has been given to the brain vascular and glial cells, particularly at high resolutions.^9^ In fact, the visualization of these cells was mostly performed with diffraction-limited optical techniques, such as epifluorescence and confocal microscopy. Brain and gut barriers are increasingly recognized as playing an active role in health and diseases by participating in whole-body responses and dynamic interactions with their environment, far beyond the concept of representing simple physical fences.^9^^–^^11^ Barriers are usually studied as a whole, with very little consideration for region-specific differences in morphology and function across the brain and the gut. Super-resolution microscopy, or optical nanoscopy, tools give unprecedented access to nanostructures that cannot be distinguished with conventional microscopy techniques (Fig. 1).^12^^–^^14^ These measurements are necessary for characterizing in detail subcellular morphological structures and gaining mechanistic insights into the barrier functional properties. Optical nanoscopy can thus complement anatomical and morphological studies performed with other imaging modalities.

**Fig. 1:**
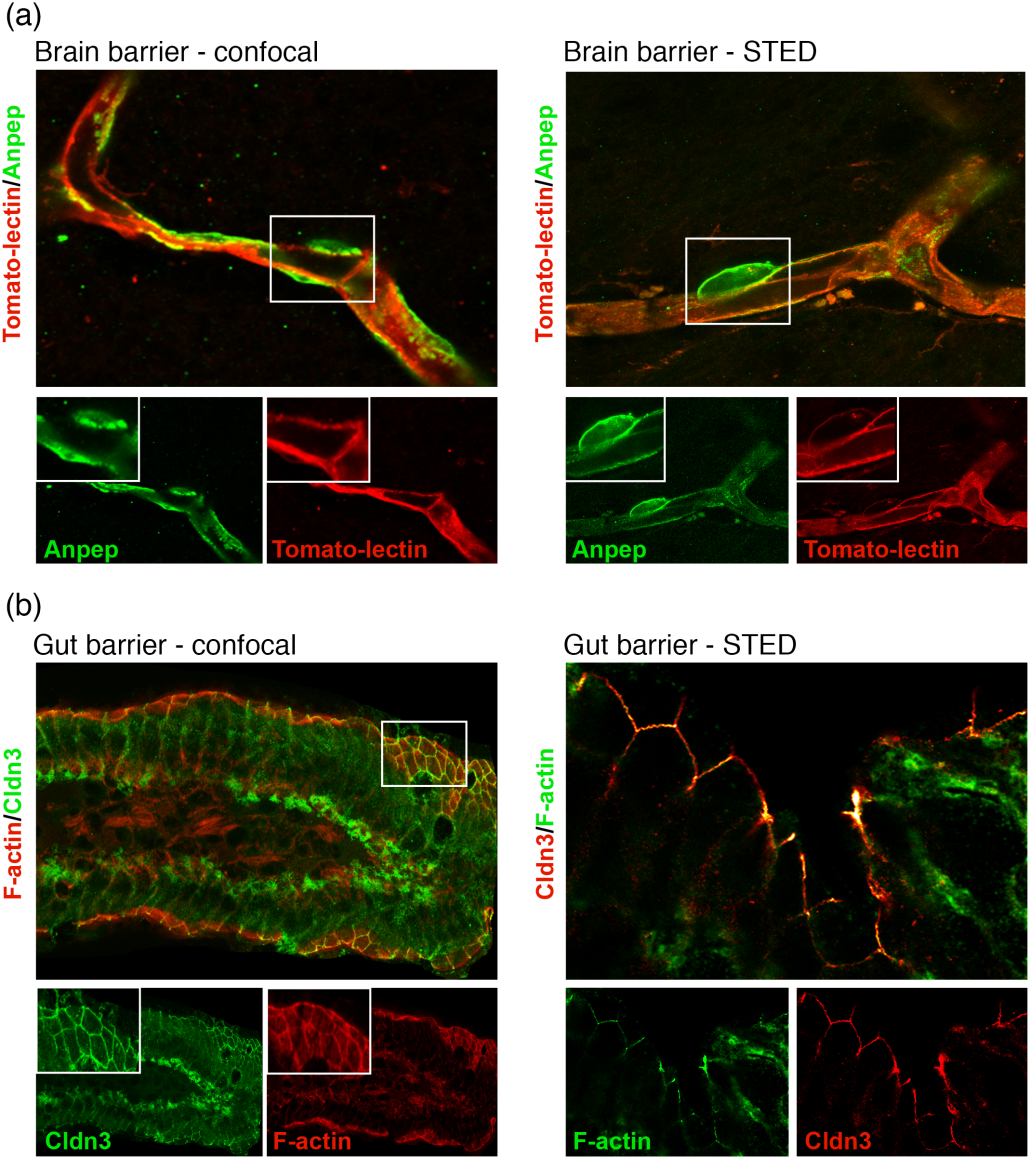
Comparison of brain and gut barrier immunostaining imaged with confocal versus super-resolution microscopy. (a) Blood vessel filled with the dye tomato-lectin tagged with Alexa-Fluor 594 and double stained with Anpep, a marker of pericytes. STED provides better resolution for evaluating physical interactions between endothelial cells forming the blood vessels and coverage by pericytes. (b) Morphological properties of the gut barrier tight junction Claudin-3 (Cldn3); for example, ruffles can be observed with STED but not confocal microscopy. Double staining was performed with F-actin to allow for the visualization of the overall structure of this organ. All experimental procedures were approved by the animal care and use committee of Université Laval (2022-1061) and met the guidelines set out by the Canadian Council on Animal Care.

Integration of cutting-edge neurophotonic tools in a project is not without challenges.^14^ As an example, in the neurovascular field, more work has to be done to decipher how different cell types interact with each other to modulate blood–brain barrier integrity and neurovascular coupling to maintain the central nervous system homeostasis.^9^ Fine mapping of cellular and sub-cellular structures with stimulated emission depletion (STED) or stochastic optical reconstruction microscopy (STORM), two super-resolution microscopy techniques, revealed a strand-like shape for Aquaporin 4 (Aqp4), a water channel localized at the astrocyte endfeet, or new structural and functional properties of the blood–brain barrier tight junctions,^4^ whereas conventional confocal microscopy only provides a gross overlap of these proteins on markers of blood vessel endothelial cells.^15^^–^^17^ This raises questions about the interpretation of previously published data and our knowledge on the function of these important components of the neurovascular unit. It is worth mentioning that not all fluorescent markers can be used for super-resolution microscopy; for example, techniques such as STORM require a blinking fluorophore, whereas for STED the fluorophores need to be depleted by a high intensity laser beam. To optimize the spatial resolution and signal-to-noise-ratio in STED microscopy, fluorophores must be bright and photostable. Aberrations can be induced by the biological specimen or optical properties—wavelength, coverslip thickness, focal plane depth, and matching refractive index between the lens immersion media and specimen—affecting image acquisition quality.^18^ Another challenge is the low expression of barrier-related proteins at baseline or under healthy conditions. Astrocyte-related structural glial fibrillary acidic protein (Gfap), S100 calcium binding protein (S100b), or Aqp4 each stain specific compartments of the cell, with differential expression between brain areas. Antibodies need to be highly specific; moreover, the number of proteins tags can be limited by the different hosts available or the number of spectrally discernible imaging channels on the microscope. Unspecific signal, or crosstalk, between detection channels can also induce analysis bias without appropriate reference samples. These challenges in experimental design and sample preparation are exacerbated with suboptimal staining, making them less forgiving when compared with classic modalities. In contrast to glial and barrier-related cells, a large number of excellent antibodies are available for neurons (PSD95, Bassoon, etc.). Finally, the blood–brain and gut barriers are large structures with a high density of vessels and cell types. Super-resolution imaging excels in imaging very small units such as synapses or vesicles and, in the context of barriers, tight junctions,^4^^,^^19^ but it may not be well suited to image networks of barrier-related cells. Still, optical nanoscopy can be powerful for studying cell–cell connections, ligand–receptor interactions, etc.

There are a lot of advantages to using quantitative super-resolution imaging^13^ to study the blood–brain and gut barriers. The detection of subtle visual phenotypes, linked to behavioral profiles, genetic, or pharmacological manipulations, can be easily overseen by the human eye,^15^ but quantitative approaches can be designed for such complex analysis tasks.^19^ The impressive images produced by super-resolution microscopy have raised the need to develop machine-learning- (ML), and more specifically deep-learning- (DL), based algorithms to improve quantitative analysis by increasing the throughput.^20^^–^^23^ ML-based approaches can lead to unbiased and detailed features-based analysis of structures and related parameters of interest.^24^^,^^25^ However, although ML strategies provide unprecedented possibilities for quantitative bioimaging, they come with several layers of complexity in their implementation, especially for users that are not versed in coding. Recently, multiple user-friendly platforms have emerged, enabling efficient sharing of trained models, reproducible DL algorithms, and more accessible analysis tools.^26^^–^^29^ This can lead to multidisciplinary collaboration opportunities between labs and trainees in biomedical sciences, neuroscience, biology, physics, microbiology, biophysics, computer science, etc. It diversifies individual skills and toolboxes as long as communication is encouraged, and a common ground is reached to define the needs and most appropriate approach in line with the scientific question. It is essential to establish an efficient analysis pipeline to use ML techniques that are suitable for small datasets, which is particularly important for animal and human studies. Importantly, data scientists can provide microscopists with tools to facilitate the annotating task, and conversely, the microscopists can share with data scientists the necessary knowledge to create an adequate annotation and training pipeline for the developed algorithms. It is also crucial to account for the variance generally observed in biological specimens even within the same group or condition. Ultimately, the amount of data generated (up to terabytes daily) can create a bottleneck for image upload, storage, transfer, processing, and analysis, and proper resources need to be allocated.

To summarize, with the right biological question in mind, super-resolution microscopy can be a powerful tool for exploring barrier-related properties and mechanisms. With the recent progress made in the development of ML approaches for optical microscopy,^30^ we can expect novel quantitative analysis strategies to be proposed for the characterization of the blood–brain barrier at the nanoscale. It should allow for the study of specific mechanisms and pathways (receptors, connexins, ligands, etc.) and will undoubtedly help biologists to identify novel barrier-related mechanisms in the healthy brain or diseases. It can also be used for projects centered on real-time imaging of living samples with the possibility of fast scanning to avoid photobleaching or photodamage.^25^ On the other hand, super-resolution microscopes are not accessible in every institution, and their operation generally requires trained experts. Additionally, references (for example, from EM) to compare obtained measurements of sub-cellular morphology and localization are not always available. Finally, increased optimization steps for sample preparation (fixation, type of antibody, concentration, etc.) and microscopy parameters can be a burden for non-experts. Therefore, concerted efforts between microscopists, biologists, and data scientists will be required to increase the application fields and democratize quantitative super-resolution microscopy. Experimental protocols should be carefully planned to improve time and cost efficiency. A thorough optimization of the acquisition parameters will also be required to enable 3D measurements with isotropic sub-diffraction resolution, which is often needed in the barrier field.
